# Supplementary material for: Characterization of BioID tagging systems in budding yeast and exploring the interactome of the Ccr4-Not complex
Source: G3 (Bethesda). 2024 Sep 13;14(11):jkae221. doi: 10.1093/g3journal/jkae221 (PMC11540327; doi:10.1093/g3journal/jkae221)
Supplement: jkae221_Supplementary_Data [file jkae221_supplementary_data.zip › Supplemental_Figures_G3-2024-405310.pdf]

Supplemental figures

A.

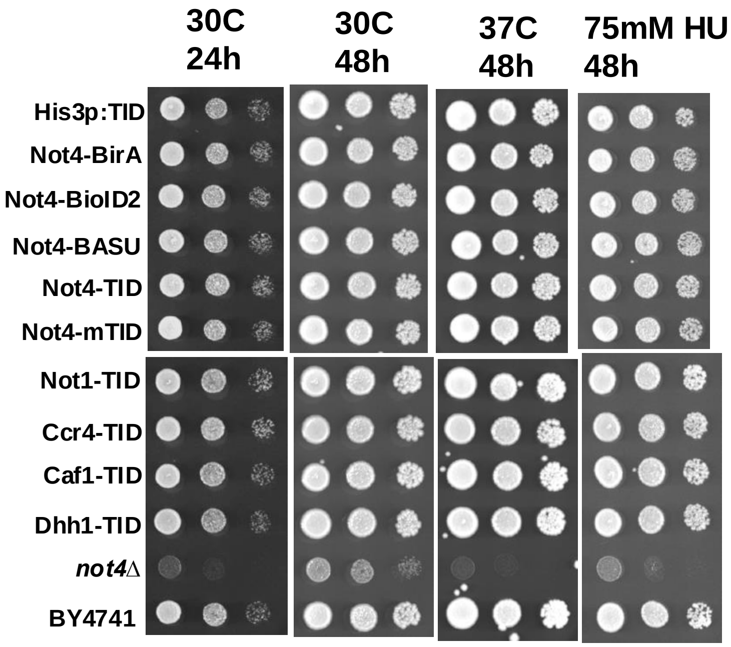
30C
24h
30C
48h
37C
48h
75mM HU
48h
His3p:TID
Not4-BirA
Not4-BioID2
Not4-BASU
Not4-TID
Not4-mTID
Not1-TID
Ccr4-TID
Caf1-TID
Dhh1-TID
not4Δ
BY4741

B.

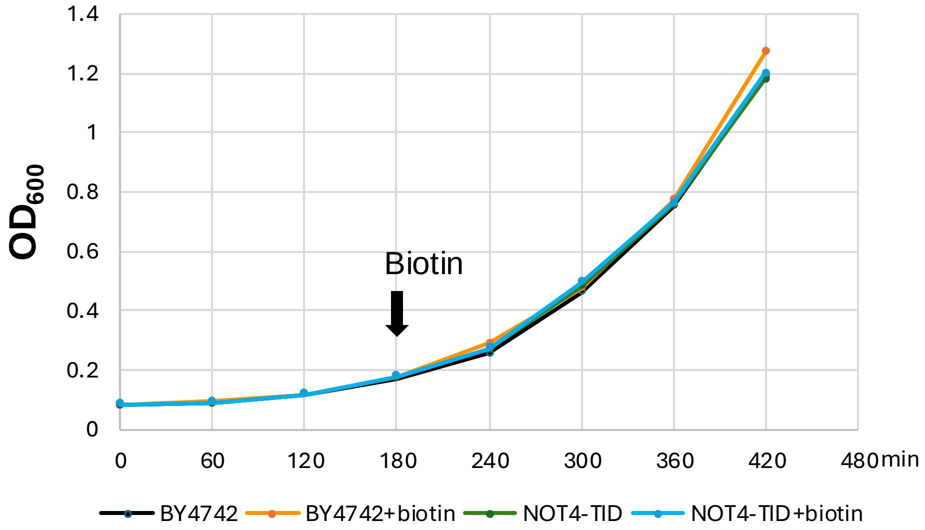
1.4
1.2
1
0.8
0.6
0.4
0.2
0
OD600
Biotin
0
60
120
180
240
300
360
420
480
min
BY4742
BY4742+biotin
NOT4-TID
NOT4-TID+biotin

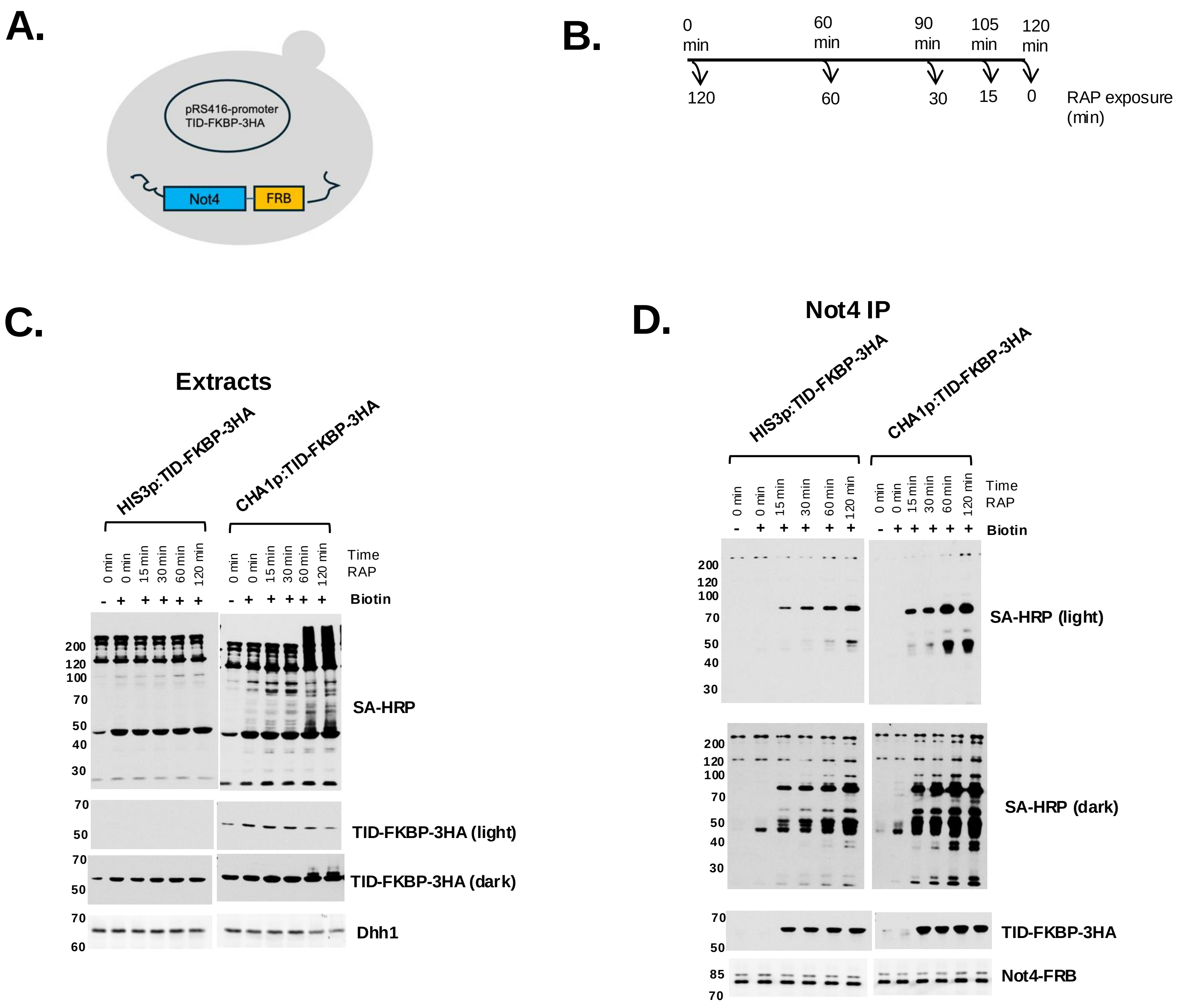
A.
pRS416-promoter
TID-FKBP-3HA
Not4
FRB
B.
0 min
60 min
90 min
105 min
120 min
120
60
30
15
0
RAP exposure (min)
C.
Extracts
HIS3p:TID-FKBP-3HA
CHA1p:TID-FKBP-3HA
0 min
0 min
15 min
30 min
60 min
120 min
Time RAP
Biotin
200
120
100
70
50
40
30
SA-HRP
TID-FKBP-3HA (light)
TID-FKBP-3HA (dark)
60
Dhh1
D.
Not4 IP
SA-HRP (light)
SA-HRP (dark)
TID-FKBP-3HA
85
Not4-FRB

Figure S2

Figure S3

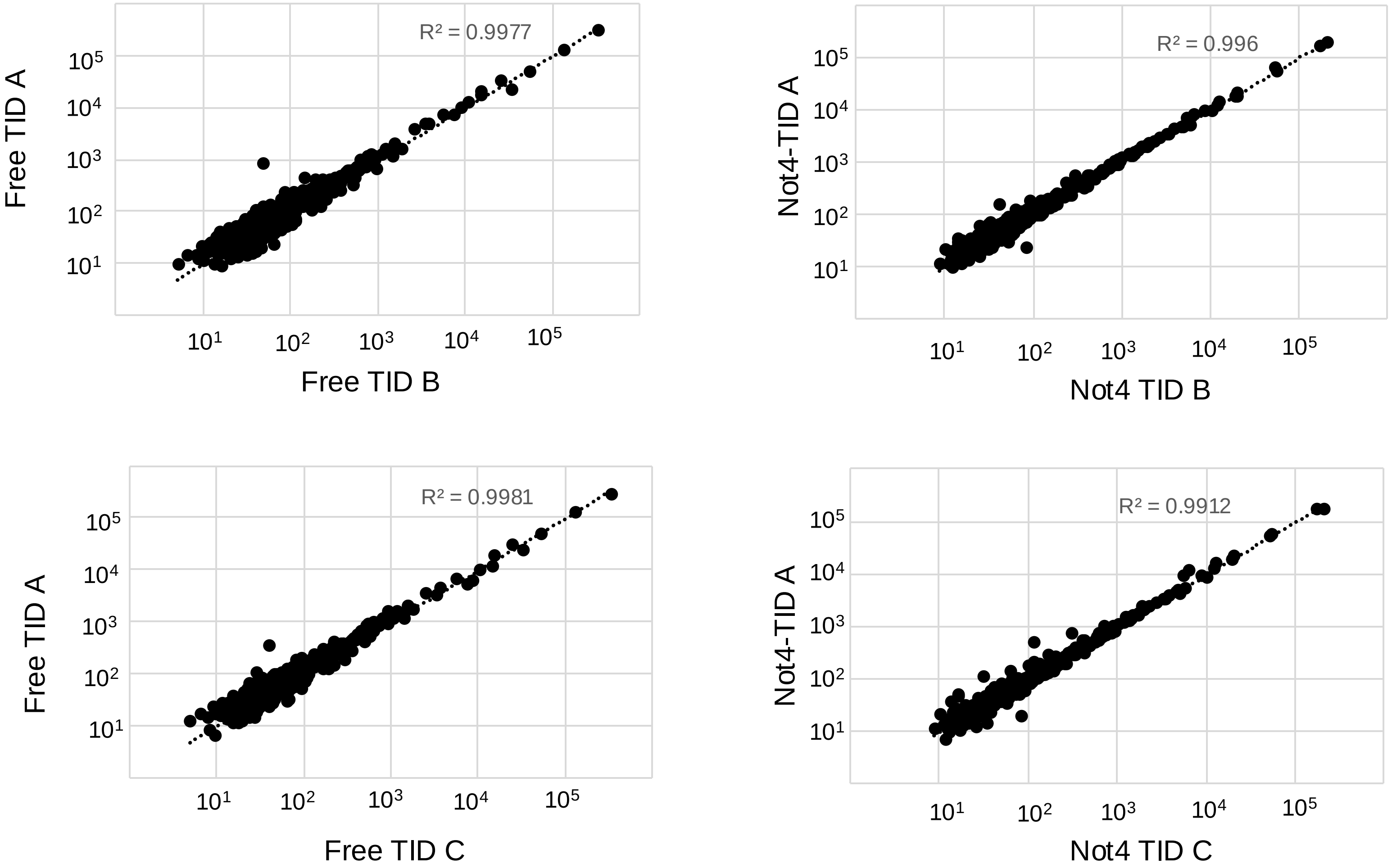

R^2 values between replicates

	Free TID	Not4 TID
A vs B	0.9977	0.996
A vs C	0.9981	0.9912
B vs C	0.9992	0.9968

Figure S4

A.

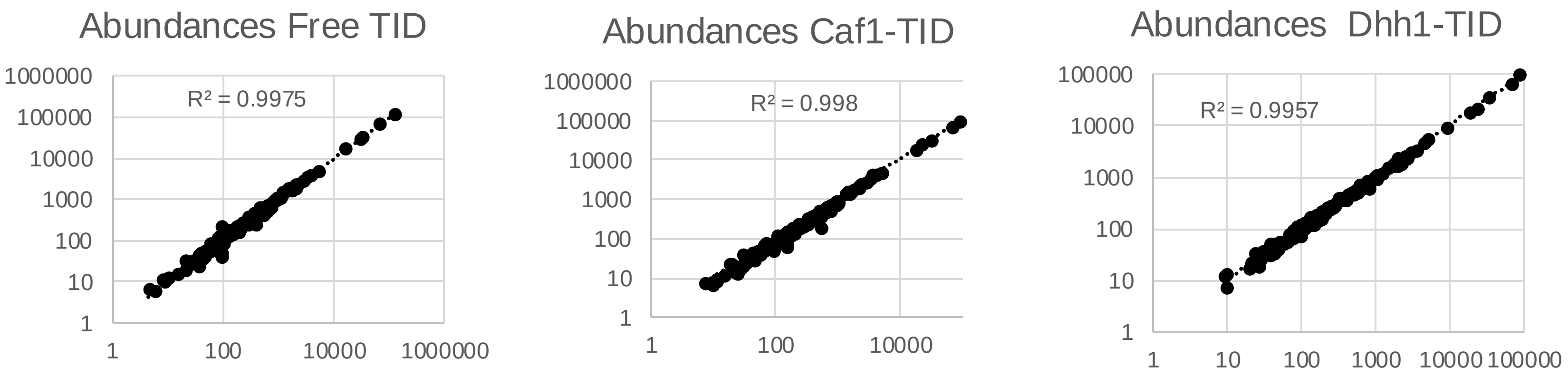

Abundances Free TID
R² = 0.9975
1000000
100000
10000
1000
100
10
1
1
100
10000
1000000
Abundances Caf1-TID
R² = 0.998
1000000
100000
10000
1000
100
10
1
1
100
10000
Abundances Dhh1-TID
R² = 0.9957
100000
10000
1000
100
10
1
1
10
100
1000
10000
100000

B.

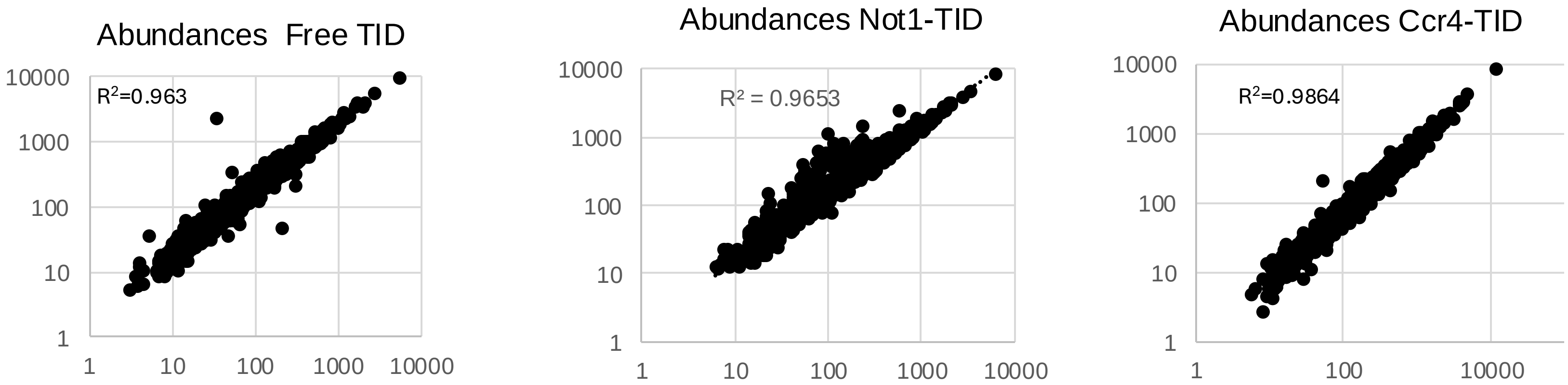

Abundances Free TID
R²=0.963
10000
1000
100
10
1
1
10
100
1000
10000
Abundances Not1-TID
R² = 0.9653
10000
1000
100
10
1
1
10
100
1000
10000
Abundances Ccr4-TID
R²=0.9864
10000
1000
100
10
1
1
100
10000

Figure S5

NOT1 enriched proteins

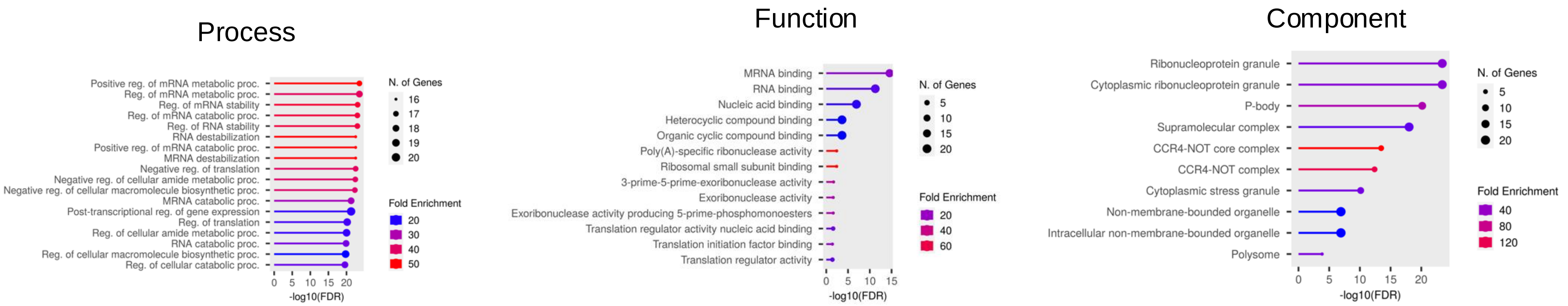

CAF1 enriched proteins

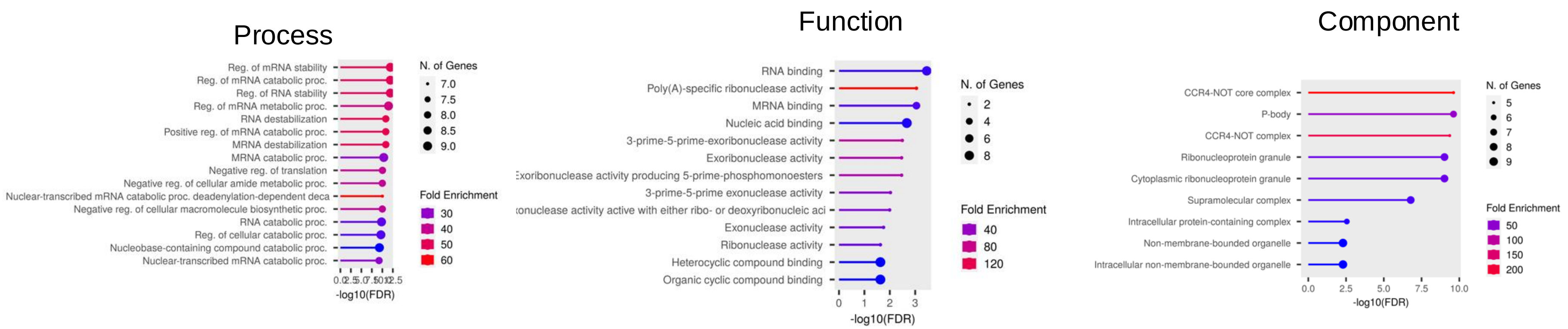

Figure S6

CCR4 enriched proteins

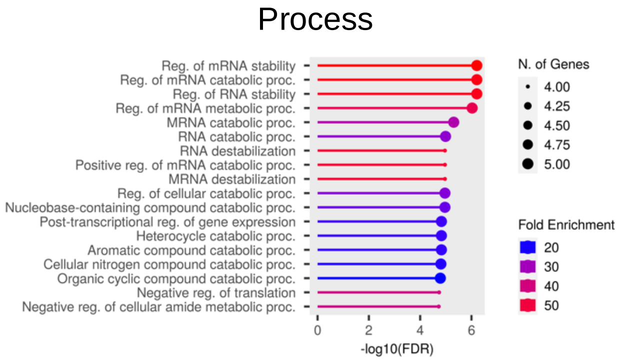

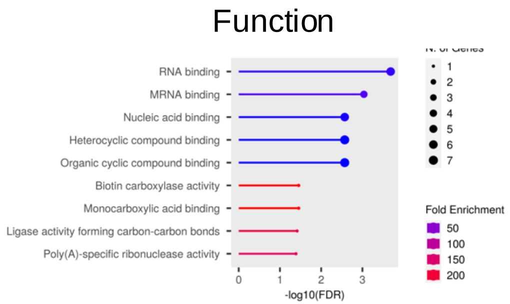

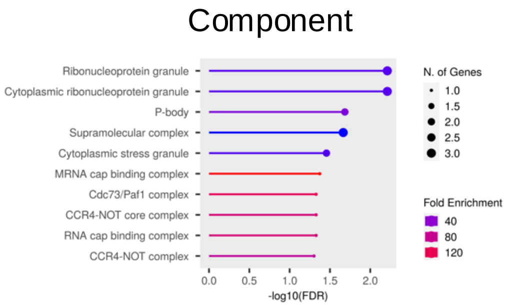

DHH1 enriched proteins

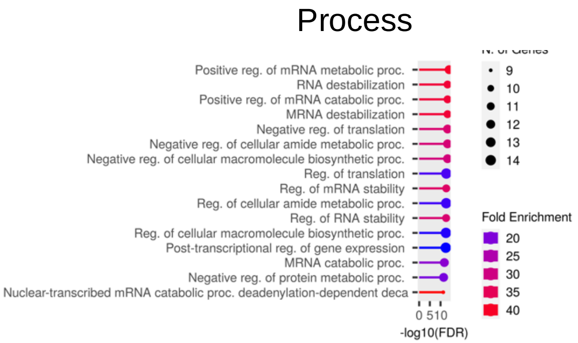

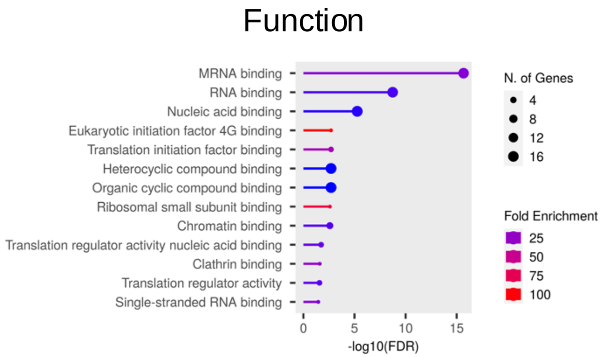

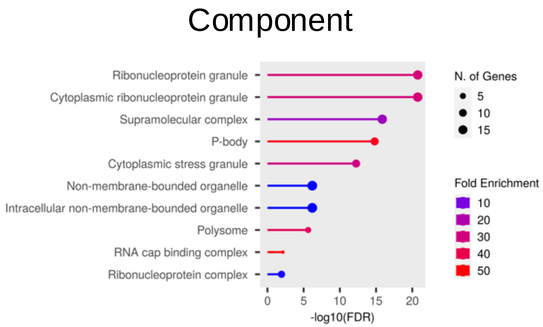

Figure S7

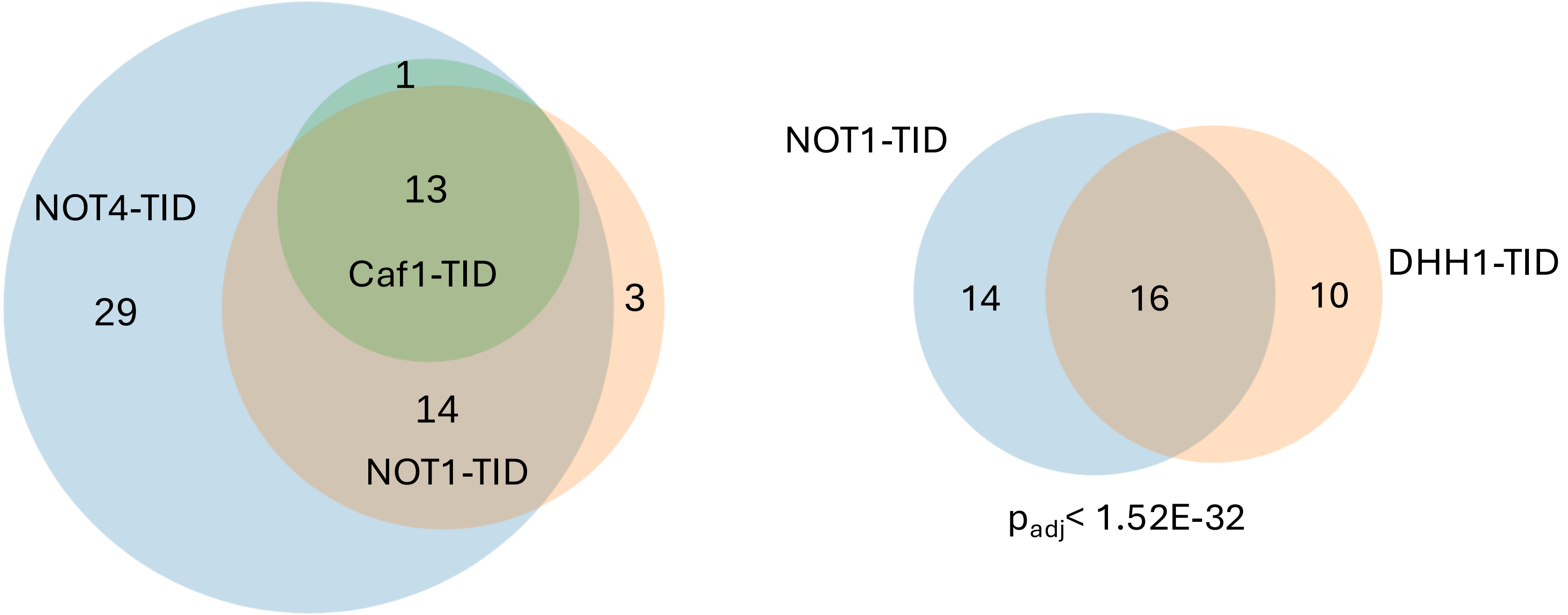

NOT4-TID
29
1
13
Caf1-TID
14
NOT1-TID
3
NOT1-TID
14
16
10
DHH1-TID
p_{adj}< 1.52E-32
