# Supplementary material for: Characterization of BioID tagging systems in budding yeast and exploring the interactome of the Ccr4-Not complex
Source: G3 (Bethesda). 2024 Sep 13;14(11):jkae221. doi: 10.1093/g3journal/jkae221 (PMC11540327; doi:10.1093/g3journal/jkae221)
Supplement: jkae221_Supplementary_Data [file jkae221_supplementary_data.zip › Supplemental_Figure_Legends_G3-2024-405310.pdf]

Supplemental figure legends

Figure S1. Spot test and growth curves of BioID strains. (A) Cells were grown to saturation in YPD and then diluted to an OD600 1.0, 0.1 and 0.01. Cells were spotted onto YPD or YPD=75 mM hydroxyurea (HU). The plates were incubated for the time indicated above the panels. Not1 is an essential gene, and deletion of Ccr4-Not subunits or *DHH1* causes slow growth, temperature, and HU sensitivity. A *not4*Δ strain is analyzed as a control. **(B).** BY4742 (unmodified strain) and NOT4-TID strains were grown to saturation in synthetic complete media plus dextrose, diluted to an OD600 of 0.075 in the same media and grown at 30˚C. Growth was monitored by recording the OD600 every hour. Biotin was added to 10 uM once the cultures started dividing at 180 min.

Figure S2. The plasmid-based CID-BioID system. (A). Plasmid-based system. A strain expressing Not4 fused to FRB [14]was transformed with a plasmid expressing FKBP-TID-HA3 from different yeast promoters. (B). Schematic of cell treatment regimen. Cells were grown in -uracil media and treated with 1uM biotin and 1 ug/ml rapamycin (RAP) as indicated in panels B and C. All cells were treated with biotin for a total of 2 hrs and the length of rapamycin treatment varied. (C). Blotting of extracts. Strains expressing TID-FKBP-3HA from the *HIS3* and *CHA1* promoter. (C). Immunoprecipitation of extracts using anti-NOT4 antibodies. The gel used to separate Not4 from the heavy chain was run on longer.

Figure S3. Scatter plot of peptide abundances between replicates from the NOT4-TID BioID experiment.

Figure S4. Scatter plot of peptide abundances between replicates from the Ccr4-NOT subunit BioID experiment.

Figure S5. GO terms of NOT1- and CAF1-enriched proteins. The collection of enriched protein genes was analyzed by ShinyGo.

Figure S6. GO terms of Ccr4- and Dhh1-enriched proteins. The collection of enriched protein genes was analyzed by ShinyGo.

Figure S7. Venn diagram of the overlap of protein-protein interactions. (A) Overlap of Not4-, Caf1- and Not1-TID enriched proteins. (B). Overlap between Not1- and Dhh1-TID.
