# Supplementary material for: Characterization of BioID tagging systems in budding yeast and exploring the interactome of the Ccr4-Not complex
Source: G3 (Bethesda). 2024 Sep 13;14(11):jkae221. doi: 10.1093/g3journal/jkae221 (PMC11540327; doi:10.1093/g3journal/jkae221)
Supplement: jkae221_Supplementary_Data [file jkae221_supplementary_data.zip › Supplemental__File_1_G3-2024-405310.docx]

**Supplemental Table 1 Yeast strains**

| BY4741 | MAT a his3Δ1; leu2Δ0; met15Δ0; ura3Δ0 |  |
| --- | --- | --- |
| BY4742 | MAT alpha his3∆1; leu2∆0; lys2∆0; ura3∆0 |  |
| JR1189 | BY4741, not4::KanMx |  |
| JR1925 | BY4742, NOT4-BirA-3HA::KanMx |  |
| JR1926 | By4742, NOT4-BioID2-3HA::KanMx |  |
| JR1927 | BY4742, NOT4-BASU-3HA::KanMx |  |
| JR1928 | BY4742, CCR4-BirA-3HA::KanMx |  |
| JR1929 | BY4742, CCR4-BioID2-3HA::KanMx |  |
| JR1930 | BY4742, CCR4-BASU-3HA::KanMx |  |
| JR1931 | BY4742, NOT4-TID-3HA::KanMx |  |
| JR1932 | BY4742, NOT4-mTID-3HA::KanMx |  |
| JR1933 | BY4742, CCR4-TID-3HA::KanMx |  |
| JR1934 | BY4742, CCR4-mTID-3HA::KanMx |  |
| JR2140 | BY4742, CAF1-TID-3HA::KanMx |  |
| JR2142 | BY4742, DHH1-TID-3HA::KanMx |  |
| JR1949 | BY4742, TEF1p-TID-3HA::KanMx |  |
| JR1950 | BY4742, HSP150p-TID-3HA::KanMx |  |
| JR1951 | BY4742, CHA1p-TID-3HA::KanMx |  |
| JR1952 | BY4742, CUP1p-TID-3HA::KanMx |  |
| JR1953 | BY4742, MET3p-TID-3HA::KanMx |  |
| JR1954 | BY4742, HIS3p-TID-3HA::KanMx |  |
| JR1963 | BY4742, NOT1-TID-3HA::KanMx |  |
| JR1918 | MAT alpha tor-1: fpr1:: LoxP-KILEU2-LoxP  ade2-11; his3-11,15; leu2-3,112; ura3-1; trp1-1; can1-100 |  |
| JR1919 | MAT alpha tor-1: frp1::NatMX; NOT4-FRB::KanMX  ade2-11; his3-11,15; leu2-3,112; ura3-1; trp1-1; can1-100 |  |
| JR1966 | MAT alpha tor-1: fpr1:: LoxP-KILEU2-LoxP;  HIS3pr-TID-FKBP-3HA::HIS3Mx  ade2-11; his3-11,15; leu2-3,112; ura3-1; trp1-1; can1-100 |  |
| JR1967 | MAT alpha tor-1: fpr1:: LoxP-KILEU2-LoxP  HIS3pr-TID-FKBP-NLS-3HA::HIS3Mx  ade2-11; his3-11,15; leu2-3,112; ura3-1; trp1-1; can1-100 |  |
| JR1968 | MAT alpha tor-1: fpr1:: LoxP-KILEU2-LoxP  CUP1pr-TID-FKBP-3HA::HIS3Mx  ade2-11; his3-11,15; leu2-3,112; ura3-1; trp1-1; can1-100 |  |
| JR1969 | MAT alpha tor-1: fpr1:: LoxP-KILEU2-LoxP  CUP1pr-TID-FKBP-NLS-3HA::HIS3Mx  ade2-11; his3-11,15; leu2-3,112; ura3-1; trp1-1; can1-100 |  |
| JR1970 | MAT alpha tor-1: fpr1:: LoxP-KILEU2-LoxP;  HIS3pr-TID-FRB-3HA::KanMx  ade2-11; his3-11,15; leu2-3,112; ura3-1; trp1-1; can1-100 |  |
| JR1971 | MAT alpha tor-1: fpr1:: LoxP-KILEU2-LoxP  HIS3pr-TID-FRB-NLS-3HA::KanMx  ade2-11; his3-11,15; leu2-3,112; ura3-1; trp1-1; can1-100 |  |
| JR1972 | MAT alpha tor-1: fpr1:: LoxP-KILEU2-LoxP  CUP1pr-TID-FRB-3HA::KanMx  ade2-11; his3-11,15; leu2-3,112; ura3-1; trp1-1; can1-100 |  |
| JR1973 | MAT alpha tor-1: fpr1:: LoxP-KILEU2-LoxP  CUP1pr-TID-FRB-NLS-3HA::KanMx  ade2-11; his3-11,15; leu2-3,112; ura3-1; trp1-1; can1-100 |  |
| JR1978 | MAT alpha tor-1: fpr1:: LoxP-KILEU2-LoxP; NOT4-FRB-KanMx  HIS3pr-TID-FKBP-3HA::KanMx  ade2-11; his3-11,15; leu2-3,112; ura3-1; trp1-1; can1-100 |  |
| JR1979 | MAT alpha tor-1: fpr1:: LoxP-KILEU2-LoxP; NOT4-FRB-KanMx  CUP1pr-TID-FKBP-3HA::HIS3Mx  ade2-11; his3-11,15; leu2-3,112; ura3-1; trp1-1; can1-100 |  |
